# Integrated analysis of single-cell and bulk RNA sequencing data reveals an immunostimulatory microenvironment in tumor thrombus of osteosarcoma

**DOI:** 10.1038/s41389-023-00474-2

**Published:** 2023-05-27

**Authors:** Tao Ji, Qianyu Shi, Song Mei, Jiuhui Xu, Haijie Liang, Lu Xie, Tingting Ren, Kunkun Sun, Dasen Li, Xiaodong Tang, Peng Zhang, Wei Guo

**Affiliations:** 1 Department of Musculoskeletal Tumor, People’s Hospital, Peking University, Beijing, 100044 China; 2 Shanghai Institute of Immunology, Shanghai Jiao Tong University School of Medicine, Shanghai, China; 3 Department of Pathology, People’s Hospital, Peking University, Beijing, 100044 China; 4 Beijing Key Laboratory for Genetics of Birth Defects, Beijing Pediatric Research Institute, MOE Key Laboratory of Major Diseases in Children, Rare Disease Center, Beijing Children’s Hospital, Capital Medical University, National Center for Children’s Health, Beijing, 100045 China

**Keywords:** Cancer microenvironment, Sarcoma

## Abstract

Tumor thrombus of bone sarcomas represents a unique reservoir for various types of cancer and immune cells, however, the investigation of tumor thrombus at a single-cell level is very limited. And it is still an open question to identify the thrombus-specific tumor microenvironment that is associated with the tumor-adaptive immune response. Here, by analyzing bulk tissue and single-cell level transcriptome from the paired thrombus and primary tumor samples of osteosarcoma (OS) patients, we define the immunostimulatory microenvironment in tumor thrombus of OS with a higher proportion of tumor-associated macrophages with M1-like states (TAM-M1) and TAM-M1 with high expression of CCL4. OS tumor thrombus is found to have upregulated IFN-γ and TGF-β signalings that are related to immune surveillance of circulating tumor cells in blood circulation. Further multiplexed immunofluorescence staining of the CD3/CD4/CD8A/CD68/CCL4 markers validates the immune-activated state in the tumor thrombus samples. Our study first reports the transcriptome differences at a single-cell level between tumor thrombus and primary tumor in sarcoma.

## Introduction

Osteosarcoma (OS) is the most common primary bone malignancy that frequently occurs in children, adolescents, and young adults [1, 2]. OS usually arises in the metaphysis of lone bones (such as the femur, the tibia, and the humerus) that have extensive longitudinal bone growth [3]. Multimodal treatment incorporating surgical resection and combination chemotherapy notably improves patients’ prognosis and has become the standard treatment of OS [4, 5]. However, nearly 20% of OS patients are found to have metastasis at diagnosis, with a poor prognosis due to insensitivity to standard therapy [2, 5, 6]. One aggressive characteristic of primary OS is its tendency to intravasate into the adjacent veins and generate a metastatic tumor thrombus (almost 23% patients of with primary pelvic OS were found to have tumor thrombus), which could be exploited as a special study model for the early stage of tumor metastasis [7]. The occurrence of tumor thrombus substantially increases the rate of metastasis in many tumor types including osteosarcoma, renal cell carcinoma, and hepatocellular carcinoma [7–9]. Patients with tumor thrombus in OS generally had a worse prognosis due to early distant metastasis and high local recurrence risk since scraps of tumor thrombus could skip across the reactive zone encapsulating the tumor and metastasize to distant organs early in the disease process [7].

Current studies about metastatic tumor thrombus mainly focus on carcinoma (including renal cell carcinoma and hepatocellular carcinoma) and analyze tumor genomic progression mostly on the tissue level [10, 11]. Through whole-exome sequencing (WES) and bulk RNA-sequencing (RNA-seq), tumor thrombus in carcinoma was found to have higher genomic instability and distinct tumor microenvironment compared to the primary tumor. Recently, Ma et al. revealed that tumor thrombus in clear cell renal cell carcinoma had more tissue-resident CD8^+^T cells and a relatively immunostimulatory state compared to primary tumor [12]. However, studies analyzing the single-cell level of transcriptome discrepancy between tumor thrombus and primary tumor are scarce and all limited to carcinoma, leading to the vacancy for the field of transcriptome difference between tumor thrombus and primary tumor in osteosarcoma.

In recent years, immunotherapy has been deemed a monumental breakthrough in oncology and become a promising therapeutic strategy for many tumor types [13, 14]. Prior studies focusing on the immune landscape of OS have shown that the tumor microenvironment (TME) of primary and metastatic OS lesions are both immunosuppressive, which is in favor of the application of immunotherapy in OS [15–17]. With heterogeneous tumor microenvironment and highly variable immune inhibitory molecule expression, however, the efficacy of immunotherapy is currently not encouraging in OS treatment [15, 18, 19]. Therefore, there is an urgent need to unveil the TME and the paradigm of immune infiltration in OS, especially the metastatic phenotype. In this study, we conducted WES, bulk RNA-seq, and scRNA-seq of paired thrombus and primary tumor samples of the OS to identify the unique features of the thrombus microenvironment, aiming at revealing the dynamic change of TME during tumor metastasis in the early stage and the transcriptome differences between tumor thrombus and primary tumor in OS.

## Results

### Molecular signature and transcriptome differences between primary tumor and tumor thrombus of human osteosarcoma

**Fig. 1:**
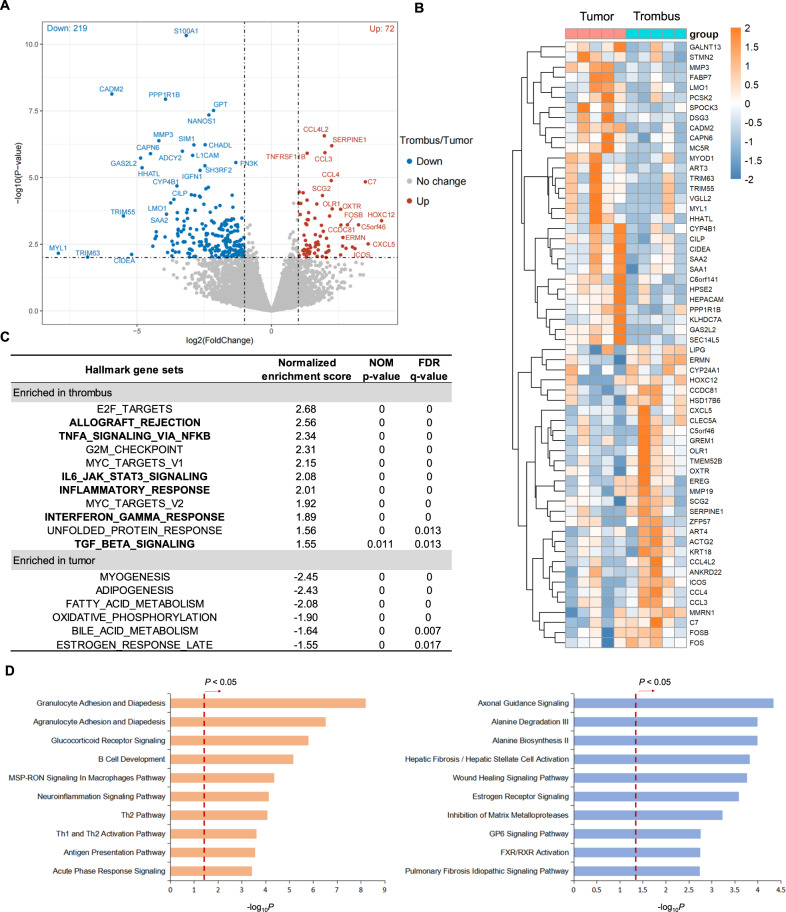
Transcriptome difference based on bulk RNA-seq data between tumor thrombus and primary tumor region of human osteosarcoma. **A** Volcano plot of differentially expressed genes in tumor thrombus compared with primary tumor. **B** Heatmap of top 30 up-regulated genes and down-regulated genes in tumor thrombus, respectively. **C** Hallmark gene sets were analyzed by GSEA. Gene sets related to immune response are shown in bold. **D** Top 10 most significant IPA canonical pathways enriched by up-regulated and down-regulated genes in tumor thrombus, respectively.

To gain insights into the molecular difference underlying tumorigenic action of primary tumor and thrombus of human osteosarcoma, we analyzed differential gene expression patterns between paired primary tumor and thrombus samples of osteosarcoma (*N* = 5) based on the RNA-seq approaches (Fig. S1). In total, 72 genes were identified as significantly up-regulated (Fig. 1A), which include many genes (including *OLR1*, *ICOS*, and *SERPINE1*) that previous reports have demonstrated their correlation with tumor cell migration and metastasis. More interesting, we found many immune-related genes (including *CCL3*, *CCL4*, *CCL4L2*, and *CXCL5*) enriched in the thrombus samples of osteosarcoma (Fig. 1B), which indicates that multiple genes involved in immunological pathways may be activated concordantly. Thus, we performed GSEA by using the MSigDB hallmark gene sets (Fig. S2) to check whether specific gene sets were correlated with the thrombus of human osteosarcoma, the GSEA enrichment results revealed that a large number of immune-related gene sets were positively enriched in samples of tumor thrombus when compare with primary tumor samples, such as the get set of “HALLMARK_INFLAMMATORY_RESPONSE”, whose expression is connected to genes defining comprehensive inflammatory response. Notably, genes involved in the “HALLMARK_TGF_BETA_SIGNALING” (genes up-regulated in response to the ligand of the TGF-beta superfamily of proteins) and “HALLMARK_INTERFERON_GAMMA_RESPONSE” (genes up-regulated in response to Interferon-gamma) were also highly positively enriched in samples of tumor thrombus (Fig. 1C). Furthermore, we employed the IPA approach to determine whether the identified differentially expressed genes could be mapped to specific canonical pathways and signal networks. As shown in Fig. 1D, “Granulocyte/Aranulocyte Adhesion and Diapedesis”, “Glucocorticoid Receptor Signaling”, “B Cell Development” and other 6 pathways consist of the most significant 10 canonical pathways of up-regulated genes in tumor thrombus. On the other hand, down-regulated genes are enriched in “Axonal Guidance Signaling”, “Alanine Degradation/Biosynthesis”, “Hepatic Fibrosis / Hepatic Stellate Cell Activation” and other 6 pathways. In summary, our IPA-based analysis generated complex functional networks and identified the possible transcription regulators that mediated inflammatory response, tissue morphology and organismal injury.

### Cell typing of the microenvironment in tumor thrombus of human osteosarcoma based on single-cell transcriptomes

**Fig. 2:**
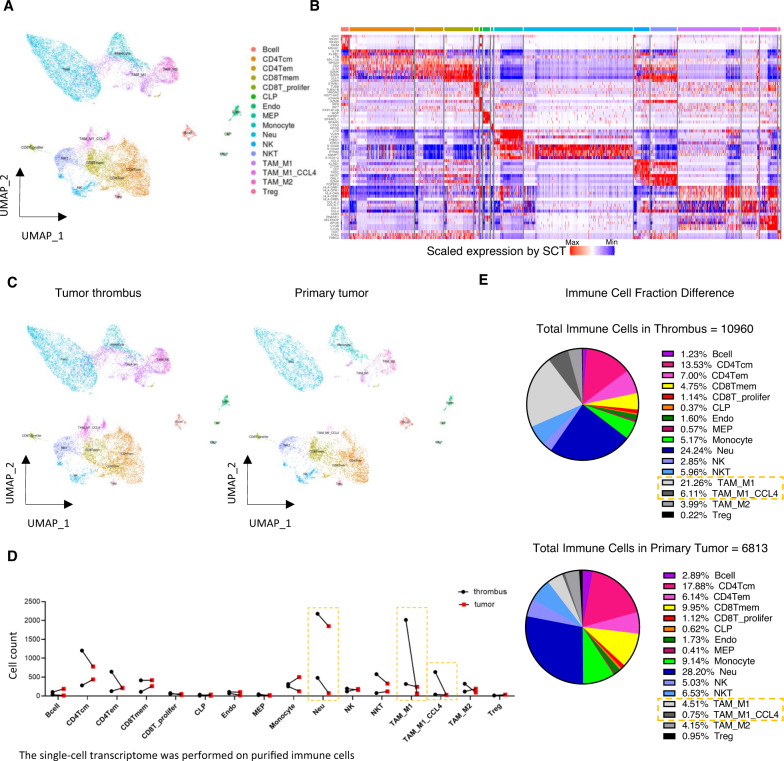
Single-cell transcriptome profile of the microenvironment in tumor thrombus of human osteosarcoma. The single-cell transcriptome was performed on purified immune cells. **A** UMAP plots of single-cell transcriptomes showing major cell types that are distinguished by different colors. **B** Heatmap of the expression value of canonical marker genes for each cell type. **C** UMAP plots of single-cell transcriptomes split by the tumor thrombus and primary tumor region. **D** Count of different clusters of cells identified by the scRNA-seq method. **E** Pie charts of the immune cell’s proportion in tumor thrombus and primary tumor. Cell types whose number or proportion is remarkably higher in tumor thrombus compared with primary tumor are signed by dotted boxes. CD4Tcm: CD4 + T central memory cells, CD4Tem: CD4 + T effector memory cells, CD8Tmem: CD8 + T memory cells, CD8T_prolifer: CD8 + T proliferating cells, CLP Common lymphoid progenitor, Endo Endothelial cells, MEP Megakaryocyte–erythroid progenitors, Neu Neutrophils, TAM_M1 Tumor-associated macrophage M1, TAM_M1_CCL4 Tumor-associated macrophage M1 with high expression of CCL4, TAM_M2 Tumor-associated macrophage M2, Treg CD4 + T regulatory cells.

To investigate the specific immune phenotypes difference between the tumor thrombus and primary tumor samples at a higher resolution, we performed the scRNA-seq of the paired primary tumor and thrombus samples of osteosarcoma (*N* = 2) with comprehensive infiltrated immune cell profiling (Fig. S1). Fresh biopsies, which were collected during surgery, were rapidly digested into single-cell suspensions, and a droplet-based scRNA-seq approach (Chromium Single-Cell Gene Expression Kit) was employed. After quality control filtering to remove cells with a small number of detected genes and high mitochondrial gene coverage, we compiled a unified cell-by-gene expression matrix of ~20,000 cells from 4 primary tumor and thrombus samples of osteosarcoma. As shown in Fig. 2A, 16 major cell clusters were identified by unsupervised dimensionality reduction and graph-based clustering analysis. Then, we annotated the cell clusters based on their top 5 marker genes to identify infiltrating immune cells, which include B cells, CD4Tcm, CD4Tem, CD8Tmem, CD8T_prolifer, CLP, Endo, MEP, Monocyte, Neutrophils, NK cells, NKT cells, TAM_M1, TAM_M1_CCL4, TAM_M2 and Treg (Fig. 2B). To identify the thrombus-specific cell-level differences compared with the primary tumor, clustering results based on UMAP renderings were split into subgroups of thrombus and primary tumor (Fig. 2C).

### Identification of immunostimulatory phenotypes in tumor thrombus of osteosarcoma

Then, we compared the cell number and immune cell fraction to gain insights into the immune phenotypes difference underlying the functional diversity of the tumor microenvironment between the tumor thrombus and primary tumor samples. As shown in Fig. 2D**and** Fig. S3, the paired comparison of cell numbers revealed the microenvironment of tumor thrombus had a generally higher cell count of the myeloid cell populations (including the Neutrophils, Tumor-associated macrophage M1, Tumor-associated macrophage M1 with high expression of CCL4). Furthermore, the cell fraction of Tumor-associated macrophage M1 and Tumor-associated macrophage M1 with high expression of CCL4 in the tumor thrombus showed about 5 fold (and 8 fold, respectively) more abundance than that in the tumor microenvironment of the primary tumor (Fig. 2E). Several pieces of evidence had pointed to an important role for the myeloid cell in the metastasis process of osteosarcoma. It is now well established that tumor-infiltrating myeloid cells are highly malleable based on the surrounding microenvironment, and an altered myeloid compartment likely contributes to the exclusion and suppression of lymphocytes. Previous reports discovered that the T cell and M1 macrophage signatures present at the pulmonary metastases interface of osteosarcoma were dwarfed by the overwhelming accumulation of immunosuppressive myeloid cells throughout the entire tumor. In conclusion, these results indicate distinct tumor microenvironment patterns between the tumor thrombus and primary tumor samples of human osteosarcoma, reflecting the tumor thrombus had a more immunostimulatory microenvironment when compared with primary tumor samples of osteosarcoma.

### Experimental validation of immunostimulatory microenvironment in tumor thrombus of osteosarcoma

**Fig. 3:**
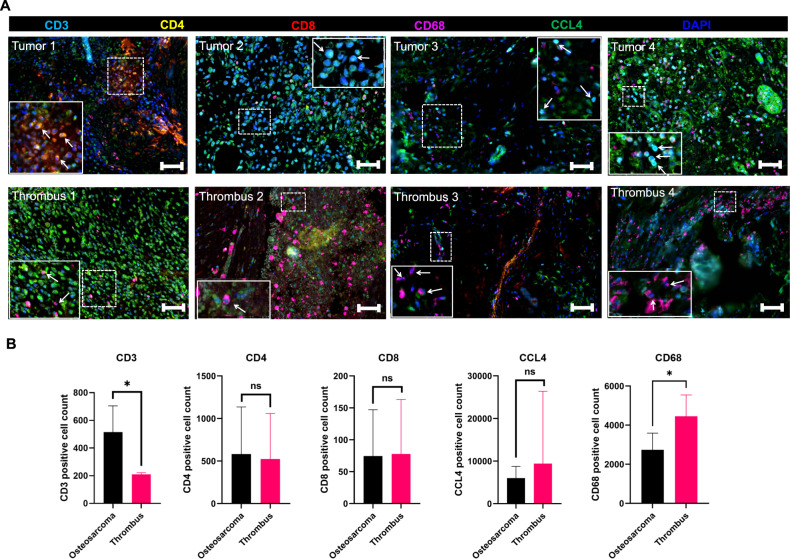
Multiplexed immunofluorescence (mIF) staining verified the immunostimulatory state in the tumor thrombus of OS. **A** mIF staining of CD3/CD4/CD8A/CD68/CCL4 markers revealed that tumor thrombus had more CD68+ macrophages expression while primary tumor had more CD3 + T cells. **B** Statistical analysis of each marker expression in primary tumor and tumor thrombus.

**Fig. 4:**
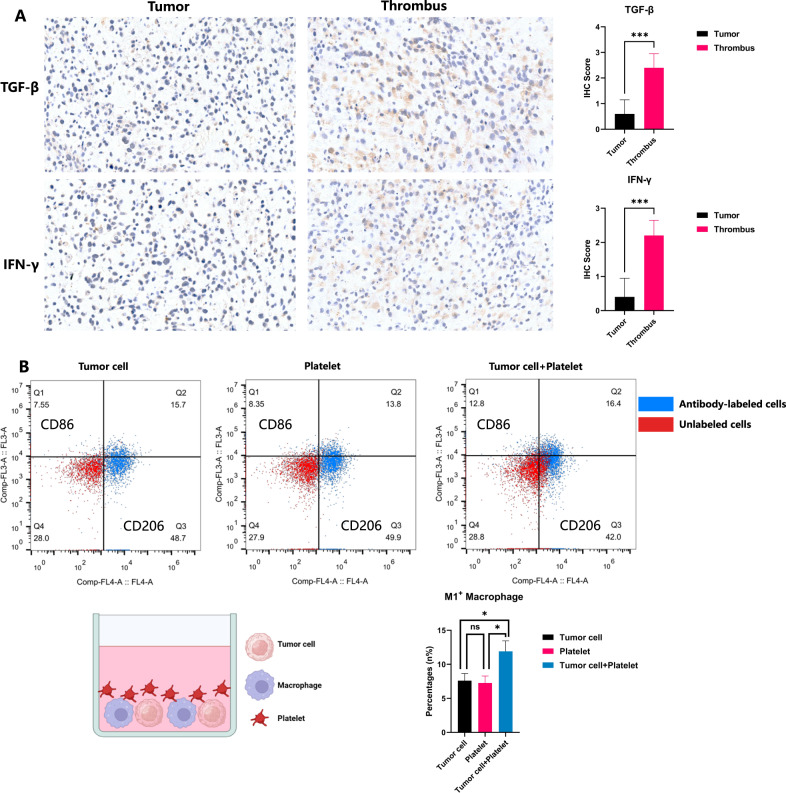
Verification of the distinct tumor microenvironment of tumor thrombus in comparison to primary tumor. **A** Immunohistochemistry (IHC) revealed upregulated expressions of TGF-β and IFN-γ in tumor thrombus tissue. **B** Flow cytometry showed increased polarization of THP-1 derived macrophages towards M1^+^ macrophage (CD86) following co-culture with tumor cells and platelets.

We further confirmed the immunostimulatory microenvironment in tumor thrombus of OS using multiplexed immunofluorescent staining (Fig. 3A). As shown in Fig. 3B, the tumor thrombus of OS had more CD68^+^ macrophages expression while CD3^+^ T cells were more abundant in the primary tumor lesions. Although the absolute cell counts of CD4^+^ and CD8^+^ T cells were approximative in tumor thrombus and primary tumor, the primary tumor had relatively higher proportions of CD4^+^ and CD8^+^ T cells. Immunohistochemistry (IHC) staining further confirmed that tumor thrombus had higher level of TGF-β and IFN-γ (Fig. 4A). Flow cytometry was performed to explore the polarization state of macrophages and the result showed an increased polarization to M1^+^ macrophages in tumor thrombus (Fig. 4B). Collectively, these results verified our scRNA-seq findings and elaborated the TME in tumor thrombus of OS in a visualized manner.

## Discussion

OS is a highly heterogenic bone malignancy with a hematogenous metastasis tendency [2]. Although combinational therapy comprising surgical resection and chemotherapy has dramatically ameliorated the prognosis of patients with localized disease, the therapeutic progress of OS has stagnated in the past thirty years [18, 20]. Immunotherapies represented by immune checkpoint blockade have acquired considerable interest for their potency in advanced tumor treatment, including OS [20, 21]. However, OS presented limited responses to immunotherapies due to its intricate TME [22–24]. Hence, revealing the cellular landscape of immune infiltration in OS may pave the way for novel therapeutic strategies on the horizon. In this current study, we first analyzed the transcriptome differences between paired primary tumor and tumor thrombus samples of OS through bulk RNA-seq. 72 significantly up-regulated genes that are involved in tumor progression and immune response were identified. GSEA analysis suggested that compared with primary tumor samples, IFN-γ and TGF-β signalings were significantly augmented in tumor thrombus. Next, we performed scRNA-seq to depict the preliminary cellular atlas for immune cell infiltration in both primary tumor and tumor thrombus samples of OS. 16 major cell phenotypes were characterized among ~20,000 cells and the cell clusters were identified as well. Analysis of immune cell number and fraction demonstrated that tumor thrombus had higher myeloid cell infiltration (including neutrophils, TAM-M1, and TAM-M1 with high expression of CCL4), which suggested a relatively immunostimulatory TME in OS tumor thrombus [25, 26]. Based on scRNA-seq results, we conducted mIF staining of T cells (CD3, CD4 and CD8), macrophages (CD68) and CCL4, which further indicated the differential distribution of immune cells between tumor thrombus and primary tumor.

**Fig. 5:**
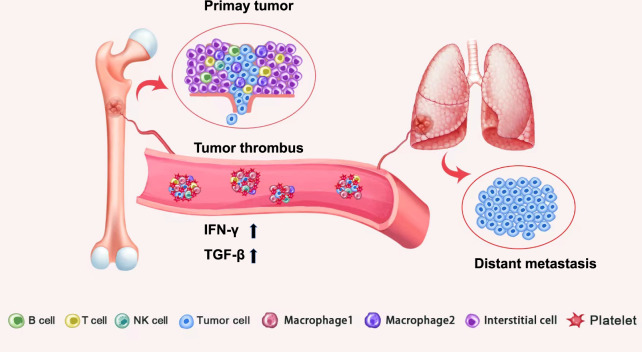
Graphic diagrams of the interplay among tumor cells, immune cells, and platelets during metastasis. Tumor cells from primary lesions invade the extracellular matrix (ECM) of primary sites and intravasate into the bloodstream, becoming circulating tumor cells (CTCs). CTCs in circulation are recognized by NK cells and initiate immune surveillance with up-regulated IFN-γ signaling. Through activating platelets to secret TGF-β, CTCs achieve immune escape and survive in the bloodstream. Meanwhile, direct contact of CTCs and platelets leads to tumor thrombus formation, which is in favor of tumor metastasis. Macrophage 1, tumor-associated macrophages with M1-like states (TAM-M1); Macrophage 2, tumor-associated macrophages with M2-like states (TAM-M2).

Previous studies about OS metastasis mainly focused on the outset and endpoint of metastasis and suggested that both primary and lung metastatic OS lesions had an immunosuppressive microenvironment accompanied by anergic anti-tumor responses [16, 17, 27, 28]. As a hallmark of hematogenous metastasis, the process of tumor cell intravasation into the bloodstream and escape from immune surveillance raised little attention in OS, and tumor thrombus could be a suitable model for analyzing this process. Therefore, we attempted to find out the unique characteristics of OS tumor thrombus TME via analysis of bulk tissue and single-cell level transcriptome from matched tumor thrombus and primary tumor samples of OS. The elevated IFN-γ signalling indicates the anti-tumor immune response in metastatic tumor thrombus. Activation of IFN-γ signaling leads to induction of the polarization of TAM to a TAM-M1 phenotype that has pro-inflammatory and tumoricidal effects [29–31]. Enriched TAM-M1 in OS tumor thrombus lesions was found based on our scRNA-seq results, which supported the bulk RNA-seq findings. Besides, IFN-γ has been reported to augment anti-tumor immune response via impeding Treg function as well as facilitating CD8^+^ T cells motility and killing capacity [32–34]. Conventionally, TGF-β signaling mainly functions as an oncogenic contributor in advanced tumors [35–37]. As a major component of tumor thrombus, platelets are the main source of TGF-β in blood circulation and TME [38]. Therefore, up-regulated TGF-β signaling in tumor thrombus may indicate the activation of platelets to counteract the local immunostimulatory TME in tumor thrombus (Fig. 5). Similar to our results, up-regulated IFN-γ signaling and antigen-presenting pathways as well as increased genes involved in immune evasion were found in renal cell carcinoma [12].

In this study, we identified an immunostimulatory TME in OS tumor thrombus with enriched TAM-M1 overexpressing CCL4 as well as elevated IFN-γ and TGF-β signalings. Our data suggested that although primary OS lesions had a highly immunosuppressive TME, anti-tumor immune response still dominates in the microenvironment of metastatic tumor thrombus. Zhou et al. has demonstrated that even in lung metastasis sites metastatic OS cells could still activate tissue-resident macrophages with a high expression level of M1 marker [17]. Being a crucial component of tumor thrombus, platelets could be activated and aggregated to assist tumor cells in evading anti-tumor immune response [39–41]. Activated platelets secreted a plethora of TGF-β to neutralize the immunostimulatory effects of IFN-γ signaling [40, 42]. The scRNA-seq and the mIF staining identified a higher amount and proportion of TAM-M1, which indicated a temporary immunostimulatory TME and represented the early stage of OS metastasis.

Our results highlight the necessity of tracking the dynamic alterations of TME during metastasis in OS. Future studies focusing on OS metastasis should (1) prospectively recruit OS patients who are complicated with both tumor thrombus and lung metastasis to collect matched primary tumor, tumor thrombus, and lung metastasis samples to investigate the TME alterations, and (2) classify immune cells with more specific markers to depict a more detailed immune microenvironment, (3) construct patient-derived xenograft mice from OS patients who are resistant to immune checkpoint therapy to explore the therapeutic effects of antiplatelet agents or TGF-β antagonists combined with immune checkpoint therapy.

In conclusion, we first reported the transcriptome differences between tumor thrombus and primary tumor in sarcoma. The current bulk tissue and single-cell level transcriptome analyses suggested an immunostimulatory microenvironment in the tumor thrombus of OS This study depicted the immune microenvironment in the early stages of OS metastasis and primed for novel therapeutic strategies for advanced OS treatment.

## Materials and methods

### Clinical specimens and patient features

Clinical specimens collected and analyzed in this study were harvested through informed consent. The study protocol was approved by the ethics committees of Peking University People’s Hospital and Beijing Children’s Hospital of Capital Medical University. The clinical characteristics of enrolled patients are listed in Table 1.

**Table 1:** Demographic and clinical characteristics of the enrolled patients.

No	Age/Sex	Diagnosis	Stage	Tumor thrombus site	Chemotherapy	Radiotherapy	Operation Time (mins)	Estimated blood loss (ml)	Follow-up time	Outcome
1	26/M	Osteosarcoma	III	Iliac vein	Yes	No	160	5000	11	DOD
2	26/M	Osteosarcoma	III	Axillary vein	Yes	No	215	900	9	DOD
3	17/F	Osteosarcoma	III	Inferior vena cava	Yes	No	230	1500	22	DOD
4	20/M	Osteosarcoma	III	Inferior vena cava	Yes	No	220	5100	20	DOD
5	24/M	Osteosarcoma	III	Iliac vein	Yes	No	225	3500	7	DOD
6	9/F	Osteosarcoma	III	Femoral vein	Yes	No	210	500	13	DOD
7	18/M	Osteosarcoma	III	Iliac vein	Yes	No	195	1500	10	DOD

*DOD* dead of disease.

### Bulk RNA-seq data analysis

We obtained clean data with the adapter removed and low-quality reads filtered. The paired-end reads were mapped to the human genome (UCSC hg38) by Hisat2 (version 2.1.0). Gene annotation and gene expression reads-count were performed by HTSeq (version 0.11.2). Then we selected the protein-coding genes and calculated the normalized expression value using the DESeq2 package (version 1.32.0) on the R platform (version 4.1.0).

### Gene-set enrichment analysis (GSEA)

The normalized expression matrix was uploaded to the gene set enrichment analysis (GSEA) software (version 4.1.0). The hallmark gene sets provided by the Molecular Signatures Database (MSigDB) were selected as a reference database. The GSEA program was run with 1000 permutations for statistical significance estimation, and the default signal-to-noise metric between the two phenotypes was used to rank all genes.

### Ingenuity pathway analysis (IPA)

Differentially expressed genes (DEGs) were analyzed by the DESeq2 package. DEGs were judged with the threshold adjusted *p*-value less than 0.05 and absolute log2 fold-change greater than 1. Then these DEGs were uploaded to the Ingenuity Pathway Analysis (IPA) software. Canonical pathways, causal networks, and upstream regulators were enriched with default parameters in this software.

### Cell proportion estimation

CIBERSORT package (version 0.1.0) in R was used to deconvolve the bulk RNAseq data. A widely used leukocyte signature matrix LM22 were used as the reference to estimate the proportion of 22 immune cell types in each sample. Then we merged some cell types based on our single-cell RNA sequencing results: “B cells naive”, “B cells memory” and “Plasma cells” were combined as “B cells”; “T cells CD4 memory resting” and “T cells CD4 memory activated” were combined as “CD4Tm”; “NK cells activated” and “NK cells resting” were combined as “NK”. Cell types which were not detected in scRNAseq and cell types estimated in less than 3 samples were removed.

### Sample collection and preparation of single-cell suspensions

Obtained during surgery, fresh tumor and thrombus tissues were stored in Dulbecco’s modified Eagle’s medium (DMEM, Gibco) with 10% fetal bovine serum (FBS, Gibco) and processed on ice. Subsequently, the tissues were rinsed with cold phosphate buffer saline (PBS, Gibco) three times and minced into ~1mm^3^ pieces. The tissue pieces were digested into single-cell suspensions using collagenase-2 (1 mg/mL) at 37 °C for 45 min and filtered through a 70-μm cell strainer. Upon digestion, the single-cell suspensions were centrifugated at 400 g for 5 min and the supernatant was discarded. Adding red blood cell lysis buffer (Solarbio) to remove red blood cells, the suspensions were then passed through a 40-μm filter and centrifugated at 500 g for 5 min, and resuspended in PBS. Cell viability was validated under the phase-contrast light microscope (Leica) after staining with trypan blue (ACMEC).

### Single-cell transcriptome library preparation and sequencing

After resection, tissue specimens were rapidly processed for single-cell RNA sequencing. Single-cell suspensions were prepared according to the protocol of Chromium™ Single Cell 3’ Solution (V2 chemistry). All specimens were washed twice with cold 1 × PBS. A hemocytometer (Thermo) was used to evaluate cell viability rates. Then, we used Countess (Thermo) to determine the concentration of the single-cell suspension and adjust the concentration to 1000 cells/μl. Samples with a cell concentration lower than that defined in the user guide (i.e., <400 cells/µl) were pelleted and resuspended in a reduced volume, and the concentration of the new solution was determined again. Finally, the cells in each sample were loaded, and libraries were constructed using a Chromium single-cell kit. Single-cell libraries were subjected to 150-bp paired-end sequencing on the Illumina NavoSeq6000 platform.

### Single-cell RNA-seq data preprocessing and quality control

After obtaining the paired-end raw reads, we used CellRanger (10x Genomics, v3.1.0) to preprocess the single-cell RNA-seq data. The cell barcodes and unique molecular identifiers (UMIs) of the library were extracted from read1. Then, the reads were split according to their cell (barcode) IDs, and the UMI sequences from read2 were simultaneously recorded for each cell. Quality control of these raw readings was subsequently performed to eliminate adapter contamination, duplicates, and low-quality bases. After filtering barcodes and low-quality readings that were not related to cells, we used STAR (version 2.5.1b) to map the clean reads to the human genome (hg19), and we retained the uniquely mapped reads for UMI counts. Next, we estimated the molecular counts and generated a UMI count matrix for each cell by counting the UMIs for each sample. Finally, we generated a gene-barcode matrix that showed the barcoded cells and gene expression counts. Based on the number of total reads, the number of detected gene features, and the percentage of mitochondrial genes, we performed quality control filtering through Seurat to discard low-quality cells (v3.1.5). Briefly, the proportion of mitochondrial genes inside one cell was calculated to be lower than 10%, and the number of total reads in one cell was below 50000. Additionally, the cells were further filtered according to the following criteria: each sample with no more than 5000 genes detected, respectively, and at least 200 genes detected per cell in any sample. Low-quality cells and outliers were discarded, and the single cells that passed the QC criteria were used for downstream analyses. Doublets were predicted by DoubletFinder (v2.0).

### Clustering analysis and cell phenotype recognition

The Seurat software package was used to perform cell clustering analysis to identify major cell types. All Seurat objects constructed from the filtered UMI-based gene expression matrixes of given samples were merged. We first applied the “SCTransform” function to implement normalization, variance stabilization, and feature selection through a regularized negative binomial model. Then, we reduced dimensionality through principal component analysis (PCA). According to standard steps implemented in Seurat, highly variable numbers of principal components (PCs) 1 to 20 were selected and used for clustering using the Uniform Manifold Approximation and Projection (UMAP). We identified the cell types of these groups based on the expression of canonical cell type markers. Finally, the 5 groups of T cells and 2 groups of ADSCs were respectively clustered for downstream analysis.

### DEG analysis

The cell-type-specific genes were identified by running Seurat with the ‘FindAllMarkers’ function on a log-transformed expression matrix with the following parameter settings: min.pct = 0.25, logfc.threshold = 0.25 (that is, there is at least a 0.25 log-scale fold change between the cells inside and outside a cluster), and only.pos = TRUE (that is, only positive markers are returned). For heatmap and violin plots, the SCT-transformed data from the Seurat pipeline were used. Using the Seurat ‘FindMarkers’ function, we found differentially expressed genes (DEGs) between the two cell types. We also used the R package clusterProfiler with the default parameters to identify gene sets that exhibited significant and consistent differences between two given biological states.

### Multiplexed immunofluorescence (mIF) staining

To validate the immunostimulatory microenvironment in the tumor thrombus of OS, mIF staining was performed using PANO 7-plex IHC kit (Cat# 0004100100, Panovue). CD3 (Cat# ab135372, Abcam), CD4 (Cat# ZM0418, ZSGB-BIO), CD8A (Cat# CST70306, CST), CD68 (Cat# CST76437, CST), CCL4 (Cat# ab235961, Abcam) and DAPI (Cat# D9542, Sigma-Aldrich) antibodies were sequentially applied, followed by horseradish peroxidase-conjugated secondary antibody incubation and tyramide signal amplification. Next, the slides were microwave heat-treated after each tyramide signal amplification operation. Nuclei were stained with DAPI after all of the antigens above had been labeled. To obtain multispectral images, the stained slides were scanned using the Mantra System (PerkinElmer), which captures the fluorescent spectra at 20-nm wavelength intervals from 420 to 720 nm with identical exposure time. 5 fields of immune cell enriched tumoral area for each slide were selected for image capture. The selected field were scanned to obtain multispectral images using the Mantra System, which captures the fluorescent spectra at 20-nm wavelength intervals from 420 to 720 nm with identical exposure time.

### Immunohistochemistry (IHC) staining

Paraffin sections of paired tumor thrombus and primary tumor underwent incubation with anti-IFN-γ (abcam, ab231036) and TGF-β (abcam, ab215715) antibodies at 4 °C overnight. Following this, the sections were visualized under a Leica microscope (Germany). Two independent pathologists, who were blinded to the clinical information of the specimens, assessed the staining scores. The staining intensity and percentage of positive cells were quantified and recorded as follows: staining intensity was graded on a scale of 0 to 3 (negative, low, moderate, and strong staining, respectively), while the percentage of positive cells was graded on a scale of 0 to 4 (0–5%, 5–25%, 25–50%, 50–75%, and >75% of total cell numbers). The weighted score for each area was calculated by multiplying the staining intensity by the percentage of positive cells. The final staining score for each case was determined by calculating the average score of 5 random areas. The grading system was based on the average scores, with grades assigned as follows: 0–1 (negative); 1–4 (positive); 4–8 (positive + ); and >8 (positive + +).

### Flow cytometry

Following two washes with phosphate-buffered saline (PBS), cells were stained with CD206 (Biolegend, 321109) and CD86 (Biolegend, 374202) antibodies in PBS containing 0.1% BSA for 30 min at 4 °C. Subsequently, the cells were subjected to flow cytometry analysis, and data were acquired and analyzed using FlowJo V10 (BD Biosciences) software.

### Quantification and statistical analysis

The specific tests used to analyze each set of experiments are indicated in the figure legends. Comparisons between two groups after IHC staining were performed using a two-tailed Student’s *t*-test, and comparisons among three or more groups were performed using one-way ANOVA. The significance of numerical values of clinical features was determined using unpaired multiple *T*-test (discovery determined using the two-stage linear step-up procedure of Benjamini, Krieger, and Yekutieli, with Q = 1%). Each row was analysed individually, without assuming a consistent SD. Numerical values were corrected for multiple comparisons using the Holm-Sidak method (alpha = 0.05, presented with padj). All statistical calculations were performed using GraphPad Prism software (ver. 7.0, GraphPad, USA) or R software (https://www.r-project.org/).

## Availability of data and materials

The single-cell sequencing and bulk RNA-seq data could be obtained from the GSA-human database (https://ngdc.cncb.ac.cn/gsa-human/) with accession number: HRA001023. The processed data and analysis codes are available upon reasonable request from the corresponding author.

## Supplementary information

Supplementary Figure 1

Supplementary Figure 2

Supplementary Figure 3
