# Supplementary material for: Integrated analysis of single-cell and bulk RNA sequencing data reveals an immunostimulatory microenvironment in tumor thrombus of osteosarcoma
Source: Oncogenesis. 2023 May 27;12(1):31. doi: 10.1038/s41389-023-00474-2 (PMC10224931; doi:10.1038/s41389-023-00474-2)
Supplement: Supplementary file 1 — Supplementary Figure 1 [file 41389_2023_474_MOESM1_ESM.pptx]

## Slide 1
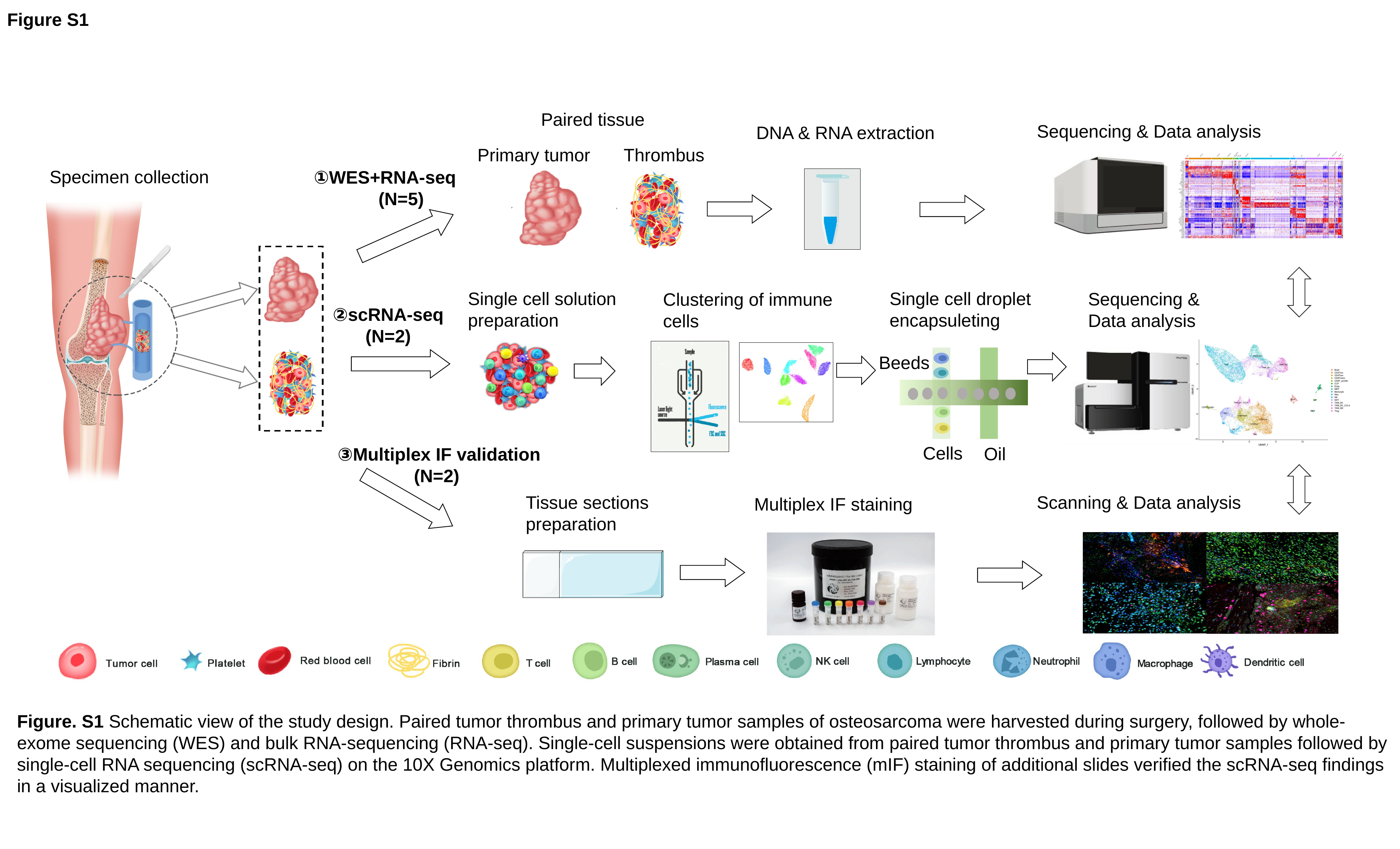

Figure S1
Paired tissue
Sequencing & Data analysis
DNA & RNA extraction
Primary tumor
Thrombus
Specimen collection
①WES+RNA-seq
(N=5)
Single cell solution
preparation
Single cell droplet
encapsuleting
Sequencing &
Data analysis
Clustering of immune
cells
②scRNA-seq
(N=2)
Beeds
Cells
Oil
③Multiplex IF validation
(N=2)
Tissue sections
preparation
Scanning & Data analysis
Multiplex IF staining
Figure. S1 Schematic view of the study design. Paired tumor thrombus and primary tumor samples of osteosarcoma were harvested during surgery, followed by whole-exome sequencing (WES) and bulk RNA-sequencing (RNA-seq). Single-cell suspensions were obtained from paired tumor thrombus and primary tumor samples followed by single-cell RNA sequencing (scRNA-seq) on the 10X Genomics platform. Multiplexed immunofluorescence (mIF) staining of additional slides verified the scRNA-seq findings in a visualized manner.
