# Supplementary material for: Integrated analysis of single-cell and bulk RNA sequencing data reveals an immunostimulatory microenvironment in tumor thrombus of osteosarcoma
Source: Oncogenesis. 2023 May 27;12(1):31. doi: 10.1038/s41389-023-00474-2 (PMC10224931; doi:10.1038/s41389-023-00474-2)
Supplement: Supplementary file 2 — Supplementary Figure 2 [file 41389_2023_474_MOESM2_ESM.pptx]

## Slide 1
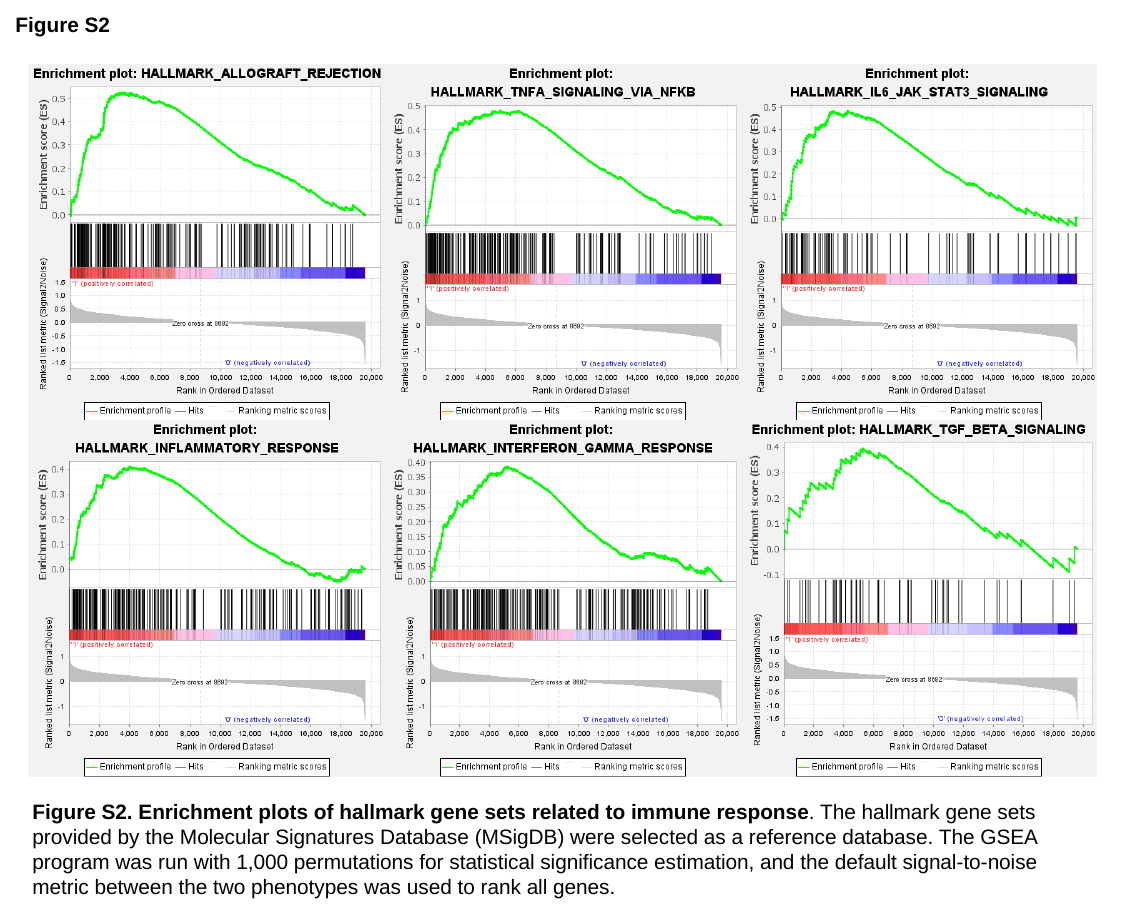

Figure S2
Figure S2. Enrichment plots of hallmark gene sets related to immune response. The hallmark gene sets provided by the Molecular Signatures Database (MSigDB) were selected as a reference database. The GSEA program was run with 1,000 permutations for statistical significance estimation, and the default signal-to-noise metric between the two phenotypes was used to rank all genes.
