# Supplementary material for: Integrated analysis of single-cell and bulk RNA sequencing data reveals an immunostimulatory microenvironment in tumor thrombus of osteosarcoma
Source: Oncogenesis. 2023 May 27;12(1):31. doi: 10.1038/s41389-023-00474-2 (PMC10224931; doi:10.1038/s41389-023-00474-2)
Supplement: Supplementary file 3 — Supplementary Figure 3 [file 41389_2023_474_MOESM3_ESM.pptx]

## Slide 1
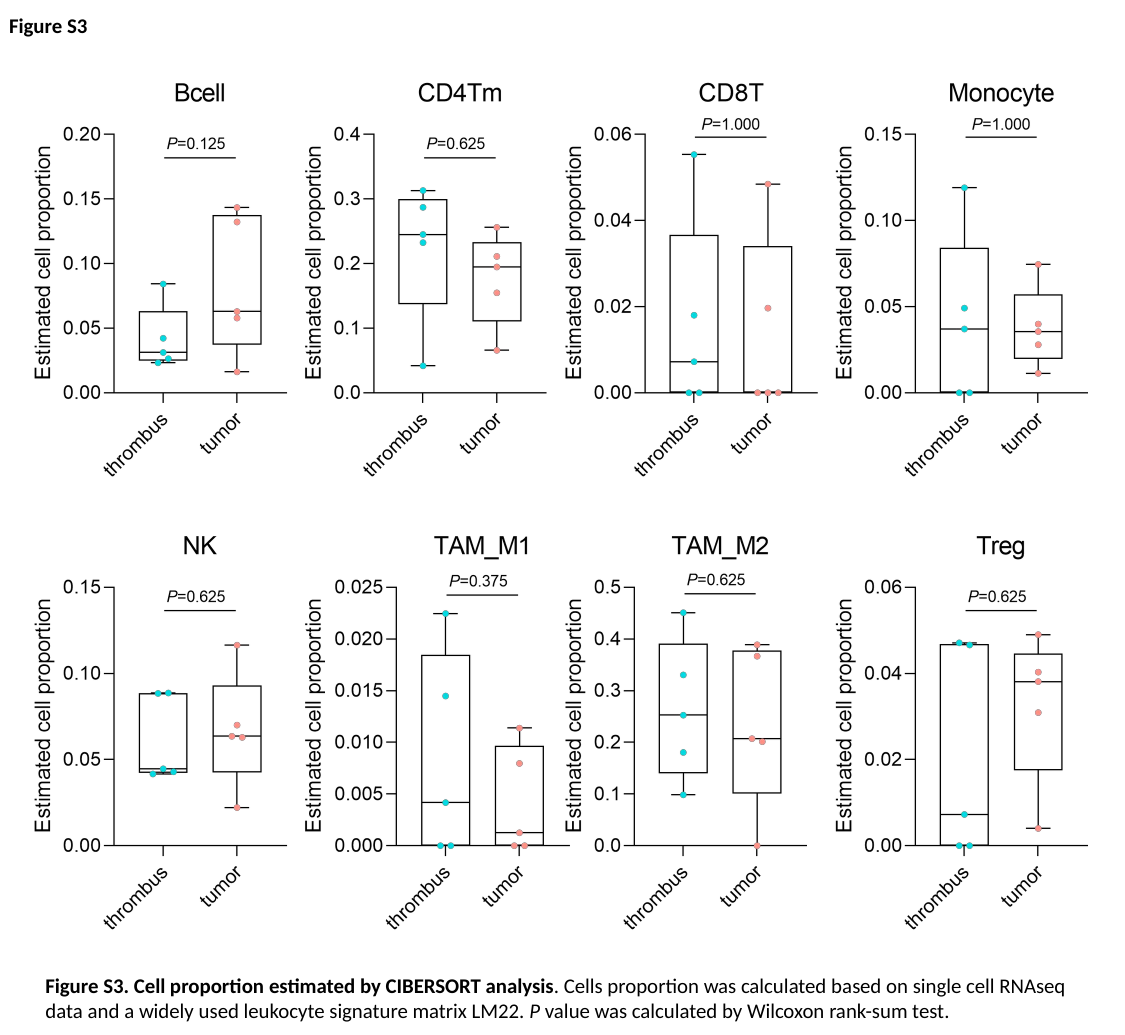

Figure S3
Figure S3. Cell proportion estimated by CIBERSORT analysis. Cells proportion was calculated based on single cell RNAseq data and a widely used leukocyte signature matrix LM22. P value was calculated by Wilcoxon rank-sum test.
